# False ground-glass opacity and suspicion of COVID-19, beware of the technique for performing the CT

**DOI:** 10.11604/pamj.supp.2020.35.138.25353

**Published:** 2020-08-09

**Authors:** Ibrahima Niang, Mame Coumba Fall, Joseph Coumba Ndoffene Diouf, Mbaye Thiam, Ibrahima Diallo, Ibrahima Faye, Sokhna Ba

**Affiliations:** 1 Radiology Department, Fann University Hospital Center, Dakar, Senegal

**Keywords:** Ground-grass opacity, CT, COVID-19, technique, deep inspiration, imaging

## Abstract

Ground-glass opacity is a CT sign that is currently experiencing renewed interest since it is very common in patients with COVID-19. However, this sign is not specific to any disease. Besides, the possibility of false positive ground-glass opacity related to insufficient inspiration during the acquisition of the chest CT should be known. We report the case of a 36-year-old patient suspected of COVID-19 and in whom a second acquisition of chest CT was necessary to remove false ground-glass opacities that erroneously supported the diagnosis of COVID-19.

## Introduction

Ground-glass pulmonary opacity is defined as a fuzzy opacity that does not erase underlying bronchial structures or pulmonary vessels on a chest CT scan [1]. This radiological sign has gained significance among clinicians and radiologists since COVID-19 pandemic. Indeed, it is recognized as the most frequent sign associated with COVID-19 infection on computed tomography [2,3]. However, this is a non-specific sign, encountered in other diseases, both benign and malignant [4,5]. In addition, this sign can realize a false positive if the technique of performing the thoracic CT is not rigorous. We report the case of a 32-year-old patient referred to the imaging department with a suspicion of COVID-19 and in whom a chest CT scan showed bilateral images of ground-glass opacity. But a second acquisition made with a blocked deep inspiration showed complete disappearance of the ground-glass opacities thus proving that they were falsely present on the first examination.

## Patients and Observation

**Figure 1:**
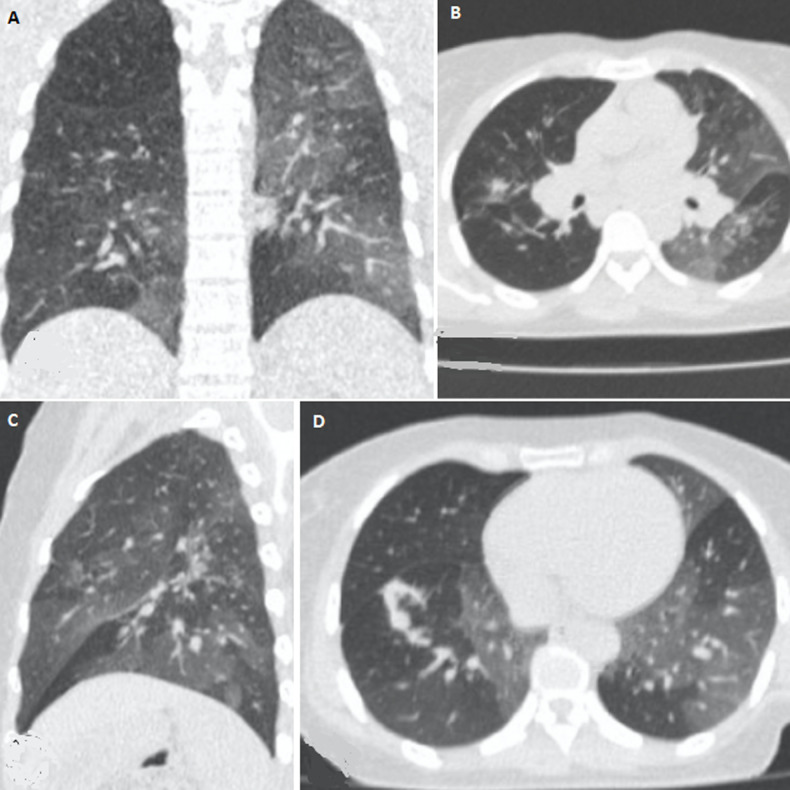
non-contrast Chest CT in lung window: A) coronal reconstruction; B) axial section; C) sagittal reconstruction showing patchy ground-glass, opacities, central and peripheral, bilateral and predominantly on the left; D) axial sections

**Figure 2:**
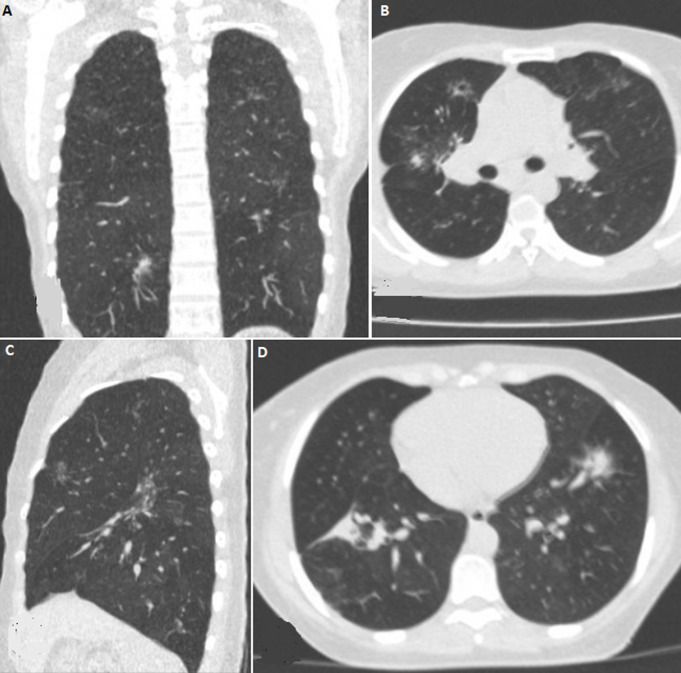
non-contrast Chest CT with deep inspiration in lung window: A) coronal reconstruction; B) axial section; C) sagittal reconstruction. Demonstrate a total disappearance of ground-glass opacities Persistence of retractile opacities; D) axial section

It was a 36-year-old female patient with no medical history, no notion of recent travel or known contact with a patient infected with COVID-19. She consulted for mild dyspnea associated with a dry cough evolving for a week without fever or other signs suggestive of COVID-19. However, with the pandemic context, she was suspected of COVID-19 and referred to the imaging department for a chest CT scan pending the availability of a PCR test. This CT scan first showed bilateral patches of ground-glass opacity in favor of the diagnosis of COVID-19 (Figure 1). The radiologist suspecting a lack in deep inspiration when performing the CT, immediately requested a second acquisition with better inspiration. This second examination showed a complete disappearance of the ground-glass opacities (Figure 2) thus proving that they were falsely present on the first acquisition. The only abnormalities finally retained on CT were retractile opacities of non-specific sequelae (Figure 2). The nasopharyngeal swab PCR test was finally performed on the patient with a negative result. She was released from the hospital with symptomatic treatment and she completely recovered a week later.

## Discussion

Chest CT is increasingly used as an additional and valuable tool in the diagnosis of patients with suspected COVID-19 [6]. Pulmonary ground-glass opacity is a sign found in between 63% and 86% of patients with COVID-19 [2,3]. This term is therefore increasingly used with COVID-19 by clinicians, although it is important to keep in mind that it is not specific to any disease [4]. Besides, it can be found in both benign and malignant conditions [5,7]. This is explained by the fact that any condition which decreases the air content of the lung parenchyma without completely obliterating the alveoli can produce ground-glass opacity [8]. This sign is easily identifiable on a chest CT in a lung window. But it can be falsely present if the patient has not taken a sufficient deep breath during the performance of CT. In our patient, with the pandemic context, the false images of ground-glass were almost wrongly associated with COVID-19 lesions. But repeating the examination with better inspiration made them disappear. This shows the importance of ensuring a rigorous technique of the chest CT, especially concerning the deep inspiration blocked at the time of acquisition. Obviously, we must take into consideration the fact that these patients, often dyspneic, do not always have the respiratory capacity allowing them to take this inspiration correctly even if it only lasts about ten seconds, the time of the acquisition. But in these patients performing prone CT is an additional trick to detect false opacity in ground glass [8].

## Conclusion

Although frequent in COVID-19 patients, the pulmonary ground-glass opacity is not a specific sign. It can be false positive especially if the deep inspiration was not carried out well at the time of the acquisition. Therefore, radiologists and technicians should ensure this technical quality and not hesitate to repeat the examination in the best conditions in case of doubt.
